# Cocaine-Induced Ascending Aortic Thrombus

**DOI:** 10.7759/cureus.47539

**Published:** 2023-10-23

**Authors:** Paul Q Vu, Siddharth Patel, Prutha R Pathak, Ashish K Basu

**Affiliations:** 1 Internal Medicine, Alabama College of Osteopathic Medicine, Dothan, USA; 2 Internal Medicine, Decatur Morgan Hospital, Decatur, USA; 3 Internal Medicine, North Alabama Medical Center, Florence, USA; 4 Cardiology, Huntsville Hospital Heart Center, Decatur, USA

**Keywords:** aortic mural thrombus, peripheral embolization, ascending aorta, acute chest pain, cocaine use

## Abstract

Aortic thrombosis without coexisting atherosclerosis is uncommon. Sometimes, aneurysms or dissections can predispose to thrombus in the abdominal or thoracic aorta. However, ascending aortic thrombus in a non-aneurysmal, non-atherosclerotic aorta is a rare occurrence. Although arterial thrombosis has been linked with its use, cocaine-associated thrombus of the ascending aorta has been rarely described. We report a young man with regular use of cocaine presenting with constant, burning, left-sided chest pain. He was found to have a large thrombus in a structurally normal ascending aorta. Medical management with therapeutic anticoagulation was started. Despite an interruption of anticoagulation treatment for two months due to non-compliance, the man survived. This unique case highlights the importance of various vascular complications associated with cocaine use, their early recognition, and their treatment.

## Introduction

Thrombosis of the aorta is usually seen in association with aneurysmal dilatation, atherosclerosis, or aortic dissection [1]. High velocity of blood flow prevents the formation of thrombus in these large-diameter aortic vessels. Another risk factor that increases the risk for an arterial thrombotic event is inherited thrombophilia (e.g., antiphospholipid syndrome, factor V Leiden mutation, prothrombin 20210, and deficiencies of protein C, protein S, or anti-thrombin) [2]. In most situations, the aortic arch, descending thoracic aorta, and abdominal aorta are the common sites affected. Thrombus formation in the ascending aorta without predisposing factors, e.g., aneurysm or severe calcification, is quite rare [3]. Cocaine abuse is rising in prevalence within the United States, and its misuse can result in a plethora of cardiovascular complications, including venous and arterial thrombosis [4]. Uncommonly, cocaine has been associated with aortic thrombosis and, in rare incidences, within the ascending aorta [5]. We report a case of a 55-year-old African American (AA) man with a history of cocaine use who presented with chest pain due to an ascending aortic thrombus.

This article was previously presented as an e-poster at the Southern Medical Association Online Conference in August 2023.

## Case presentation

A 55-year-old AA man presented to the emergency room (ER) with left-sided, constant, burning chest pain radiating to his left shoulder that started when he woke up in the morning. He reported 10/10 intensity pain without aggravating or alleviating factors. Chest pain was associated with diaphoresis, nausea, and an episode of non-bilious vomiting. The patient also admitted to using cocaine the night before the onset of symptoms. His past medical history was significant for hypertension, diabetes type 2, chronic kidney disease, and a history of deep venous thrombosis (DVT) of the lower extremities. He had a 30+ pack-year smoking history and consumed five to six alcoholic beverages daily. He had an inferior vena cava (IVC) filter placed four months prior due to chronic lower limb ischemia and medical noncompliance. He was admitted for further evaluation and treatment.

**Figure 1:**
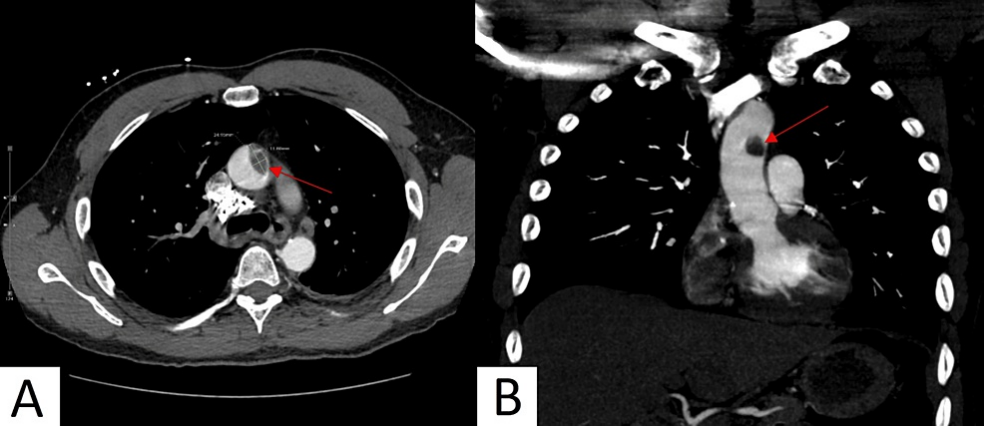
Axial (A) and coronal (B) section of CT pulmonary angiogram. Prominent irregular mural thrombus measuring 2.5 x 1.2 cm in the ascending aortic arch (red arrow).

On initial physical examination in the ER, he had a temperature of 97.9 F, a blood pressure of 182/107 mmHg, a pulse of 85 per minute, respirations of 19 per minute, and an oxygen saturation of 97% on room air. Although vital signs were stable, given the patient's past medical history and acute onset of symptoms, the differential diagnosis for his clinical presentation included pulmonary embolism (PE) and acute coronary syndrome. Auscultation of the heart and lungs revealed normal S1 and S2 without murmur, rub or gallop and normal breath sounds without wheeze, rhonchi, or crackles, respectively. Laboratory studies, complete blood count (CBC), comprehensive metabolic panel (CMP), lipase, coagulation studies, troponin, D-dimer, pro-B-type natriuretic peptide (pro-BNP), urine drug screen, and SARS-CoV-2 PCR were obtained [Table 1, 2]. The electrocardiogram (EKG) showed normal sinus rhythm with non-specific changes in ST segment and T waves and a high-sensitivity troponin series that was elevated, prompting further evaluation with an echocardiogram, which showed a 65% ejection fraction without regional wall motion abnormality. A pulmonary arteriogram study was conducted since the patient had an elevated D-dimer of 1.71 mg/mL fibrinogen equivalent units (FEU). The arteriogram showed a tiny acute PE at the right lower lobe and, incidentally, a prominent irregular, unstable thrombus in the ascending aorta. There were no signs of calcification, dissection, or aneurysm [Figure 1]. A cardiothoracic surgeon was consulted, and medical management was advised. The patient was started on an unfractionated heparin intravenous infusion according to the heparin protocol. Unfortunately, the patient left against medical advice and presented two months later with lower extremity pain. He was readmitted, and, despite interruption of anticoagulation treatment, the second pulmonary arteriogram showed persistent aortic thrombosis at the exact location as the first study, although with new embolic complications. He developed bilateral renal infarcts and dry gangrene of the second and third digits of the right foot. Imaging did not detect filling defects in renal arteries but did detect occlusions of the right superficial femoral artery and the right popliteal artery, for which he underwent atherectomy and angioplasty.

**Table 1:** Blood investigations on admission

Test:	Result:	Reference range:
White blood cell count	7.07 x 10^3 ^cells/mm3	4.8-10.8 x 10^3^ cells/mm3
Red blood cell count	5.63 x 10^6^ cells/mm^3^	4.7-6.1 x 10^6^ cells/mm^3^
Hemoglobin	15.6 g/dL	14.0-18.0 g/dL
Hematocrit	46.1%	42.0-52.0%
Mean corpuscular volume	81.9 FL	81-99 FL
Mean corpuscular hemoglobin	27.7 PG	27-31 PG
Mean corpuscular hemoglobin concentration	33.8 g/dL	33-37 g/dL
Red cell distribution width-standard deviation	13.8%	11.5-14.5%
Platelet count	243 x 10^3^ cells/mm^3^	130-400 x 10^3^ cells/mm^3^
Mean platelet volume	10.1 FL	7.4-10.4 FL
Prothrombin time	12.5 seconds	11.0-16.0 seconds
International normalized ratio	0.97	0.9-1.1
Partial thromboplastin time	26.4 seconds	22.3-41.8 seconds
D-dimer	1.71 mg/mL FEU	0.0-0.52 mg/mL FEU
Sodium	137 mmol/L	136-145 mmol/L
Potassium	4.2 mmol/L	3.5-5.1 mmol/L
Chloride	100 mmol/L	88-107 mmol/L
Carbon dioxide	21 mmol/L	25-35 mmol/L
Anion gap	16 mmol/L	8-14 mmol/L
Blood urea nitrogen	10 mg/dL	8-22 mg/dL
Creatinine	1.3 mg/dL	0.7-1.2 mg/dL
Glucose	248 mg/dL	70-104 mg/dL
Calcium	9.5 mg/dL	8.8-10.2 mg/dL
Total bilirubin	0.19 mg/dL	0.20-1.00 mg/dL
Aspartate aminotransferase	14 U/L	10-34 U/L
Alanine aminotransferase	12 U/L	10-44 U/L
Alkaline phosphatase	105 U/L	32-122 U/L
Troponin T, high sensitivity	39 ng/L (0 hour), 49 ng/L (3.5 hour), 93 ng/L (5.5 hour)	0-19 ng/L

**Table 2:** Urine drug screen & COVID-19 test on admission

Test:	Result:
Urine opiate screen	Positive
Urine oxycodone screen	Positive
Urine methadone, qualitative	None detected
Urine barbiturate screen	None detected
Urine phencyclidine screen	None detected
Urine amphetamine screen	None detected
Urine benzodiazepine screen	None detected
Urine cocaine screen	Positive
SARS-COV-2 (PCR)	Negative

## Discussion

Aortic mural thrombus (incidence of 0.45%) arising from the aorta is exceedingly rare without precipitating factors (e.g., aneurysmal dilatation, atherosclerosis, or aortic dissection) [5]. Conditions that allow thrombus formation include a hypercoagulable state, endothelial injury, and stasis of blood flow (Virchow’s triad) [3,6]. Furthermore, common sites of thrombus occurrence in the aorta include the thoracic aorta and aortic arch (74%), followed by the abdominal aorta (14%), and the ascending aorta (12%). The rarity of aortic thrombosis stems from high blood flow within larger vessels, especially at the opening of the aortic valve [1]. Although the common symptoms include limb ischemia (84%), visceral ischemia (27%), and stroke (14%), our patient initially presented in the ER with none of these symptoms and only complained of chest pain [1]. Our investigations included laboratory studies, EKG, echocardiogram, troponin, and D-dimer levels to evaluate for myocardial ischemia and pulmonary embolism. While his troponin levels were elevated, his EKG showed normal sinus rhythm with non-specific changes in ST segment and T waves, and his echocardiogram showed a 65% ejection fraction without regional wall motion abnormality. It was felt that the increase in troponins was most likely secondary to cocaine use [7]. With an elevated D-dimer, a pulmonary arteriogram was performed and showed a tiny acute PE at the right lower lobe and an irregular thrombus adherent to the ascending aorta without any significant aneurysm, calcification, or dissection. Given the location (right lower lobe) and size of the PE, it was thought unlikely to have contributed to his acute, left-sided chest pain. With the patient also having no other precipitating factors or history of thrombophilia (e.g., factor V Leiden, prothrombin 20210, protein C, protein S, anti-thrombin deficiency, antiphospholipid syndrome), it was concluded that his aortic thrombosis was a direct result of chronic cocaine use [8]. Cocaine has been demonstrated to activate platelets, facilitate alpha-granule release, and result in the formation of platelet-containing microaggregates [9]. In addition, chronic cocaine abuse results in a dysfunctional endothelium that is unable to vasodilate due to impaired nitric oxide release and an increase in vasoconstrictive factors (e.g., thromboxane A2, serotonin, and endothelin) [8,10]. As stated previously, thrombosis of the ascending aorta is not common without any precipitating factors. However, ascending aortic thrombus most commonly results in an acute myocardial infarction leading to devastating consequences (cardiogenic shock or sudden death of 39.5% and in-hospital death of 31.6%) [3]. Although mortality rates were comparable at 6.2% and 5.7% for the anticoagulation therapy and surgery, respectively (P = 0.879), there is preference for open thrombectomy (65%) over medical intervention with anticoagulation (35%) due to the reduced risk of recurrent distal embolization [1,3]. Due to concerns regarding poor compliance, our patient was treated medically with IV heparin but did not finish treatment. He presented two months later with embolic complications involving bilateral lower extremities. Interestingly, despite not being on any anticoagulation therapy, the patient fortunately survived, albeit with lower extremity ischemia from embolism.

## Conclusions

This case demonstrates an odd presentation of ascending aortic thrombosis without any precipitating factors or history of thrombophilia. Although rare, it is important to consider aortic thrombus in patients presenting with cocaine-induced chest pain, as cocaine is able to induce thrombogenicity and is not always due to coronary spasm. With respect to intervention, it is imperative to start anticoagulation therapy since delay in treatment will result in embolic complications.
